# Comparing type 1 and type 2 diabetes in pregnancy- similar conditions or is a separate approach required?

**DOI:** 10.1186/s12884-015-0499-y

**Published:** 2015-03-27

**Authors:** Lisa A Owens, Jon Sedar, Louise Carmody, Fidelma Dunne

**Affiliations:** Atlantic Diabetes in Pregnancy Programme, Galway, Ireland; Galway Diabetes Research Centre, National University of Ireland, Galway, Ireland

**Keywords:** Pregnancy, Type 1 diabetes, Type 2 diabetes

## Abstract

**Background:**

Pregnancy in women with type 1 (T1DM) or type 2 diabetes (T2DM) is associated with increased risk. These conditions are managed similarly during pregnancy, and compared directly in analyses, however they affect women of different age, body mass index and ethnicity.

**Methods:**

We assess if differences exist in pregnancy outcomes between T1DM and T2DM by comparing them directly and with matched controls. We also analyze the effect of glycemic control on pregnancy outcomes and analyze predictive variables for poor outcome.

**Results:**

We include 323 women with diabetes and 660 glucose-tolerant controls. T2DM women had higher BMI, age and parity with a shorter duration of diabetes and better glycemic control. Preeclampsia occurred more in women with T1DM only. Rates of elective cesarean section were similar between groups but greater than in controls, emergency cesarean section was increased in women with type 1 diabetes. Maternal morbidity in T1DM was double that of matched controls but T2DM was similar to controls. Babies of mothers with diabetes were more likely to be delivered prematurely. Neonatal hypoglycemia occurred more in T1DM than T2DM and contributed to a higher rate of admission to neonatal intensive care for both groups. Adverse neonatal outcomes including stillbirths and congenital abnormalities were seen in both groups but were more common in T1DM pregnancies. HbA1C values at which these poor outcomes occurred differed between T1 and T2DM.

**Conclusions:**

Pregnancy outcomes in T1DM and T2DM are different and occur at different levels of glycemia. This should be considered when planning and managing pregnancy and when counseling women.

## Background

Pregnancy for women with pre-gestational diabetes is high risk. There is an increase in morbidity for the mother and an increase in morbidity and mortality for the baby. To date many observational studies have examined pregnancy outcomes for women with type 1 and type 2 diabetes as a group [1,2]. Few studies however have compared the clinical outcomes between the two groups [3] or have compared either group to matched controls [4]. Results of studies done comparing the two groups have been conflicting. In clinical practice women with both conditions are treated in a similar manner, at the same clinics, with similar monitoring and treatment targets. However these two conditions have different pathologies, and affect two very different groups of women with different age, weight, ethnic background and duration of disease.

The aim of this retrospective case–control study was to examine pregnancy outcomes in women with type 1 diabetes and type 2 diabetes directly and compare pregnancy outcome of each group with matched normal-glucose tolerant controls. Furthermore we aimed to assess if there are specific factors that predict poor outcome and if these predictive factors are different between the two groups. Finally we sought to assess if specific HbA1C recordings in each trimester were predictive of good/poor outcome and if these HbA1C recordings are different in the two groups.

## Methods

The Atlantic Diabetes in Pregnancy (DIP) program was established in 2007 and aims to provide evidence based care for women with Diabetes Mellitus before, during and after pregnancy. It has a dual role, providing clinical care as well as undertaking observational cohort studies aiming to enhance knowledge and improve management for women with Diabetes in pregnancy. It comprises 5 centers on the Irish Atlantic seaboard, with11000 deliveries annually.

All women with type 1 or type 2 diabetes for more than six months prior to the index pregnancy were included. Only singleton pregnancies were included. Women were managed within a multi-disciplinary service and were seen every two to four weeks throughout pregnancy. The multi-disciplinary team includes consultant diabetologist, consultant obstetrician, diabetes nurse specialist, midwife with an interest in diabetes and dietitian. Pre-pregnancy care (PPC) was offered to all women. This was done by letter and/or phone call. General practitioners and practice nurses were also informed via letter of the service and invited to refer women.

Normal glucose tolerance (NGT) controls were recorded as part of a cohort study of universal screening for gestational diabetes Mellitus (GDM) [5]. They had a normal 75 g Oral Glucose Tolerance test at 24–28 weeks gestation as defined by IADPSG [6] (International Association of the Diabetes and Pregnancy Study Groups). Written consent was obtained, as was ethical approval (Galway University Hospital Research Ethics Committee, Letterkenny General Hospital Research Ethics Committee, Mayo General Hospital Research Ethics Committee, Sligo General Hospital Research Ethics Committee). Two controls were included for each index case. Body Mass Index (BMI) was recorded at the first visit and at each subsequent visit. The first visit varied from 4 to 25 weeks but the average was at 15 weeks gestation.

All demographic data, treatment and outcomes were recorded electronically. Recorded data included age, ethnic group, gravidity, parity, family history of diabetes, folic acid use, smoking status, body mass index (BMI) preeclampsia, pregnancy induced hypertension, need for neonatal unit care (NNU), polyhydramnios, neonatal hypoglycemia, jaundice or hypocalcemia, elective or emergency cesarean section, instrumental delivery, miscarriage, stillbirth and congenital malformations. Blood pressure and weight were recorded at each visit and HbA1C was recorded at the first visit, in each trimester, and prior to delivery. HbA1c was measured by reverse phase cation exchange chromatography using the Menarini HA8160 automated haemoglobin analyser. The method was calibrated according to International Federation of Clinical Chemistry (IFCC) standardisation. Results are reported in IFCC and DCCT (Diabetes Control and Complications Trial) format.

### Obstetric/perinatal outcome definition

Miscarriage was defined as pregnancy loss before viability (24 weeks gestation). Stillbirth was defined as loss of a viable fetus (after 24 weeks gestation). Preterm delivery was defined as before 37 weeks. Large for gestational age was defined as birthweight ≥ 90th centile, extreme large for gestational age as birthweight ≥ 97.7th centile and small for gestational age as birthweight ≤ 10th centile.

Gestational hypertension was defined as a blood pressure greater than 140/90, without proteinuria, on two or more occasions greater than 6 hours apart, in a woman with normal blood pressure at her first obstetric visit. Preeclampsia was defined as blood pressure greater than 140/90, with proteinuria, on two or more occasions greater than 6 hours apart after 20 weeks of gestation.

A composite of neonatal and maternal outcome was used in the statistical analysis. Maternal composite included: Preeclampsia, pregnancy-induced hypertension and emergency cesarean section. The neonatal composite included: stillbirth, miscarriage, premature delivery, polyhydramnios and neonatal hypoglycemia.

### Statistical analysis

Statistical analysis was carried out using the R program. Chi-square and independent samples *T*-test were used for comparison between type 1 and type 2 and diabetes and controls. Controls were selected from a group of over 12000 women universally screened for gestational diabetes mellitus as part of a separate trial. Those with similar age, body mass index, parity and ethnic group to the group with diabetes were used. This was completed using cosine similarity matching with a customized nearest neighbors selection without replacement. Predictive modeling was done using classification tree based supervised learning. Significance level was set at a p-value of 0.05.

## Results

### Demographics (Table 1)

**Table 1:** **Demographic data of women with type 1, type 2 diabetes and controls**

**Demographic**	**Type 1 diabetes**	**T1DM controls**	**Type 2 diabetes**	**T2DM controls**	**P value T1DM vs T2DM**
Total n = 983	215	447	108	213	
Mean age	31.9 ± 5.6	32 ± 5.7	33.7 ± 4.8	32.6 ± 4.8	0.0001
Non-Caucasian ethnicity	0.14% (n = 3)	0.13% (n = 6)	30% (n = 33)	22% (n = 45)	0.0001
Mean first BMI (kg/m^2^)	28.3 ± 4.8	28.4 ± 4.9	34.9 ± 6.7	33.2 ± 6.2	0.001
Mean gravidity	2.3 ± 1.4	2.3 ± 1.5	2.9 ± 2.3	2.7 ± 1.7	0.002
Attended pre-pregnancy care	44%	-	34%	-	0.02
Pre-pregnancy folic acid use	65%	47%	55%	43%	ns
Mean duration of Diabetes (years)	14 ± 8.2	-	4.3 ± 3.8	-	0.001

323 women, 215 with T1DM and 108 with T2DM were included in this study and matched with 660 NGT controls. All but 3 women with T1DM were of Caucasian ethnicity, whereas 33 (31%) of women with T2DM were of non-Caucasian ethnicity (two thirds of which were of Asian descent and one third black African). Women with T1DM were younger, with a lower gravidity and lower first recorded BMI and a longer duration of disease than women with T2DM. Attendance for pre-pregnancy care (PPC) occurred more commonly in women with T1DM compared to T2 DM. Folic acid was used pre-pregnancy in 65% of women with T1DM and 55% with T2DM.

### Glycemic control (Table 2)

**Table 2:** **Mean HbA1C values during pregnancy for type 1 and type 2 diabetes**

**Pregnancy stage**	**Type 1 diabetes (% ± SD/mmol/mol)**	**Type 2 diabetes (% ± SD/mmol/mol)**	**P value**
Overall mean HbA1C	6.6%/49	5.8%/40	<0.0001
Trimester 1	7.7 ± 1.6/61	6.9 ± 1.7/52	0.0007
% with HbA1C <7% in T1	41%	60%	0.024
Trimester 2	6.7 ± 1.1/50	6.1 ± 1.1/43	<0.0001
Trimester 3	6.4 ± 0.95/46	5.9 ± 0.67/41	0.0004
Term	6.4 ± 0.88/46	5.7 ± 0.45/39	0.02

Trimester specific mean HbA1C values are seen in Table 2. Overall the mean HbA1C in pregnancy is lower in women with T2 compared to T1 DM (5.8 vs. 6.6%, p = 0.001). In each trimester and prior to delivery mean HbA1C is also significantly lower in women with T2 compared to T1 DM and in both groups HbA1C improves as pregnancy progresses reaching a nadir of 6.4% and 5.7% in women with T1 and T2 DM respectively at term. Women who attended PPC in either group had a lower first trimester HbA1C compared to those who did not attend. Women who attended pre-pregnancy care had a lower mean first trimester HbA1C recorded (T1DM 7 ± 1% vs 8.4 ± 1.8%, T2DM- 7.3 ± 1.8% vs 6.4 ± 1%, p = 0.001).

### Maternal outcomes (Table 3)

**Table 3:** **Maternal and neonatal outcomes for women with type 1 and type 2 diabetes in pregnancy and controls**

**Outcome/variable** **% (n)**	**Type 1 diabetes**	**Matched control**	**P-value**	**Type 2 diabetes**	**Matched control**	**P- value**	**P value T1DM vs T2DM**
N = 983	215	447		108	213		
Pre-pregnancy folic acid	65%	47%	0.001	55%	43%	0.06	0.10
Maternal outcomes							
Preeclampsia	12% (12)	4.3%(19)	.0003	8.3% (9)	8% (17)	1	0.4
Gestational hypertension	20% / 43	10% (45)	.0003	22% (24)	11% (23)	.014	0.8
Emergency section	29% / 92	16% (72)	.0002	17% (18)	15% (32)	.86	0.08
Elective section	30% / 65	15% (67)	.002	36% (39)	19% (40)	.002	0.3
Maternal composite*	46.5%	25%	.08	38%	29%	.16	0.17
Neonatal outcomes							
Miscarriage	11%	N/A**		8.3%	N/A**		0.5
Stillbirth	2.8%(6)	0.4% (2)	.027	1.9% (2)	0.9%(2)	.86	0.5
Neonatal deaths	0	0		0	0		
Live birth rate	85.6%	99%	.0001	90%	97.7%	.012	0.25
Congenital malformation	4.2% (9)	1.6% (7)	.074	2.8% (3)	1.9% (4)	.90	0.74
Polyhydramnios	10% (22)	1.8% (8)	.001	6.5% (7)	5.6%(12)	.95	0.36
Neonatal hypoglycemia	20% (43)	0.4% (2)	.0001	6.5% (7)	0.5% (1)	0.004	0.002
Hypocalcemia	0	0.2% (1)	1	0	0	1	
Neonatal jaundice	6% (13)	5.4%(24)	0.86	8.3% (9)	4.7%(10)	0.29	
Shoulder dystocia	2.3% (5)	1.1% (5)	0.39	1% (1)	0.5% (2)	1	
Polycythemia	0.9% (2)	0%	0.19	0.9% (1)	0	0.72	
Mean (kg)	3.54 ± 0.6	3.54 ± 0.6	1	3.52 ± 0.8	3.54 ± 0.7	1	1
Weight >4 kg (%)	30%	21%	0.03	20%	24%	0.4	0.08
Weight >4.5 kg	6.5%	4%	0.27	8.2%	3.8%	0.16	0.76
Large for gestational age	24%	17%	0.07	20%	22%	0.9	1
Small for gestational age	5.6%	6.5%	0.8	5.6%	6.5%	0.9	1
Delivery < 37 weeks	28% (60)	5.4%(24)	.001	22% (24)	8.5%(18)	.001	0.29
Neonatal Unit care	55%	14%%	.0001	39%	17% (36)	.0001	0.009
Antepartum hemorrhage	0.9% (2)	3% (13)	0.14	0%	3% (6)	0.13	0.8
Postpartum hemorrhage	2.8% (6)	4.9%(22)	0.28	3.7% (4)	4.7%(10)	0.9	0.9
Neonatal composite***	48%	6.5%	.0001	32%	10%	.06	0.008

*Maternal composite: Preeclampsia, gestational hypertension, emergency cesarean section.

**N/A: Controls were screened for GDM at 24 weeks, i.e. beyond the date of possible miscarriage.

***Neonatal composite: Stillbirth, miscarriage, premature delivery, polyhydramnios, hypoglycemia.

Preeclampsia (PET) occurred more commonly in women with T1DM compared to matched controls while women with T2DM and their matched controls had a similar prevalence of 8%. On the other hand the rate of gestational hypertension (GH) was similarly increased in T1 and T2 women compared to controls. Elective cesarean section (CS) was similarly increased in women with T1 and T2 DM compared to matched controls. Emergency CS was increased only in women with T1DM. Polyhydramnios was increased only in women with T1 DM compared to controls but rates were similar in women with T2 DM compared to controls. Ante- and postpartum hemorrhage were not different between groups and controls. Overall composite maternal morbidity was twice as common in women with T1 DM but not significantly different in women with T2 DM when compared to matched controls.

### Neonatal outcomes (Table 3)

Babies born to mothers with T1DM and T2 DM were more likely to be delivered before 37 completed weeks of gestation when compared to matched controls. There was no difference in mean birth weight between groups (3.54 kg) and controls. There was an increase in babies born > 4 kg to women with T1DM compared to matched controls. Neonatal hypoglycemia was more prevalent in offspring of both T1 DM and T2 DM pregnancies when compared to matched-controls and higher in offspring of T1DM than T2DM. Hypocalcemia, neonatal jaundice, shoulder dystocia and polycythemia were similar between groups and matched controls. These however are relatively rare outcomes and the numbers in this study are too small to detect significant differences in these outcomes. The stillbirth rate was higher in babies of T1DM mothers compared to controls, but no difference was seen in mothers with T2 DM. Stillbirth occurred due to major malformation in 1 case and the results of post mortem are not recorded in our database. Congenital anomalies were higher in T1DM than controls, but the difference did not reach statistical significance (p = 0.07). Malformations that occurred were mainly classical ones seen with maternal diabetes and included; cardiac transposition (n = 3), ventricular septal defect (n = 3), atrial septal defect (n = 1), hydrocephalus (n = 1), cystic kidney (n = 1), rectal atresia (n = 1), missing tibia (n = 1), esophageal fistula (n = 1), sacral agenesis (n = 1) and caudal regression syndrome (n = 1).

There were no neonatal deaths. More babies of T1 than T2 DM mothers were admitted to the neonatal intensive care unit (NNU) compared to controls. The most common reasons for admission to NNU were hypoglycemia (29% of admissions) and prematurity (27%). Composite poor neonatal outcomes were significantly greater in offspring of T1 than T2 mothers compared to controls. Overall the live birth rate was significantly lower for babies born to women with both T1DM and T2DM compared to their matched controls and non- significantly lower in pregnancies of T1 compared to T2 women. There was no statistically significant difference in miscarriage rate between T1 and T2 women.

### Impact of glycemic control (Table 4)

**Table 4:** **Impact of glycemic control- Mean HbA1C values of pregnancy in those having poor outcomes and those without poor outcomes for type 1 and type 2 diabetes**

**Outcomes**	**T1DM-**	**T1DM-**	**T2DM-**	**T2DM-**	**P value T1DM vs T2DM poor outcome**
**Mean HbA1C ± SD %/mmol/mol**	**Poor outcome**	**No poor outcome**	**Poor outcome**	**No poor outcome**	
Gestational Hypertension	7.2% (55) ±1.2%	6.8%(51) ±1.3	6.4%(46) ±0.9	6.2%(51) ±1.1	0.027
Pre-eclampsia	7.2% (55) ±1.3	6.8% (46) ±1.3	6.3% (45) ±1	5.8% (41) ±0.6	0.003
Emergency csection	7.2% (55) ±1.3	6.8% (51) ±1.3	6% (39) ±1	6.3% (42) ±1	0.048
Elective csection	6.8% (51) ±1.1	7% (53) ±1.4	6.3% (45) ±0.8	6.3% (45) ± 1.1	0.009
Maternal composite	7.2% (55) ±1.2	6.7% (51) ±1.3	6.2% (44) ±1	6.3% (45) ±1	0.017
Neonatal Hypoglycemia	7.1% (54) ±0.9	6.3% (45) ±0.9	6.7% (50) ±1.5	5.9% (41) ±0.5	0.55
Miscarriage (trimester 1 HbA1C)	7.8% (62) ±1.2	6.8% (51) ±1.9	7.7% (61) ±1.6	6.2% (44) ±0.8	0.5
Stillbirth	8.8% (73) ±1.3	6.8% (51) ±1.3	n/a (n = 1)	n/a	n/a
Premature delivery	7.4% (57) ±1.5	6.7% (50) ±1.2	6.6% (49) ±1.4	6.2% (44) ±0.8	0.28
Polyhydramnios	7.1% (54) ±1.1	6.8% (51) ±1.3	6.7% (50) ±0.9	6.2% (44) ± 1.3	0.01
Congenital malformation (trimester 1 HbA1C)	7.1% (54) ±1.6	7.5% (58) ±1.5	6.4% (46) ±0.7	7.3% (56) ±1.8	0.38
Neonatal composite	7.3% (56) ±1.4	6.5% (47) ±1.1	6.6% (49) ±1.3	6.1% (43) ±0.7	0.034

We analyzed the effect of HBA1C values throughout pregnancy on maternal and neonatal outcomes for both types of diabetes. We report the mean HbA1C for those who did or did not have the studied poor maternal and neonatal outcomes for both T1DM and T2DM. HbA1C values were significantly higher for women with T1DM who had gestational hypertension, polyhydramnios, emergency cesarean section, elective cesarean section during their pregnancy than it was for women with T2DM who had these outcomes. Maternal and neonatal composite poor outcomes also occurred at higher HbA1C values in T1DM than T2DM.

### Predictive variables

We used classification tree analysis to predict pregnancy factors that predict poor maternal and fetal outcomes. We looked at these outcomes over the full pregnancy period and per trimester. We created a composite of poor maternal and poor fetal outcomes (see Table 3 for components of composite). In women with T1DM, predictive factors of poor maternal outcome were nulliparity, booking BMI >27 kg/m^2^ and trimester 1 HbA1C of > 7.8%/68 mmol/mol. For those with T2DM, the only predictor was a booking BMI >38 kg/m^2^ but for those with this high BMI older age (>35 years) conferred worse outcomes. Higher HbA1C did not predict poor maternal outcome for T2DM. In women with T1DM, HbA1C of >6.8%/51 mmol/mol was the only clear predictor of composite poor fetal outcome and for those with T2DM the main predictive variable was hypertension.

## Discussion

Type 1 and type 2 diabetes in pregnancy have been associated with high maternal morbidity [1,4,7,8] as well as high neonatal morbidity and mortality [9,10]. These increasingly more common conditions complicate 1-3% of all pregnancies and the prevalence is rising due mainly to an increase in the incidence of type 2 diabetes [11]. We have published our efforts [12] to improve these outcomes through pre-pregnancy care programs and intensifying glycemic control. However the outcomes of pregnancies complicated by diabetes in our cohort of predominantly Caucasian women continue to be worse than their counterparts without diabetes. In order to try and isolate areas for potential improvement we wanted to assess whether the various outcomes in pregnancy were different in those with type 1 and type 2 diabetes. We felt this to be an important step in the process of providing optimal, individualized, evidence-based care for our women throughout pregnancy as well as accurate patient counseling for our women.

We address this by several different approaches combined (a) direct comparison (b) comparison with matched controls (c) predictive modeling of poor outcomes and (d) assessing outcomes based on HbA1C values.

Several observational studies have analyzed the outcomes for type 1 and type 2 diabetes as a group, compared with controls [13,14] and in most centers and guidelines the management of diabetes during pregnancy are similar for both types [15]. As the incidence of both types of diabetes continues to increase [14]^,^ full recognition of the risks and appropriate management of both conditions are paramount. There have been several studies comparing the two conditions with each other or with matched controls to assess if outcomes are similar in order to promote similar treatment approaches, however results have been conflicting^1^ [16-19], Our paper adds to the knowledge gap by looking at a large group of predominantly Caucasian women with mainly well-controlled diabetes, who have been provided intensive monitoring during pregnancy and a significant proportion of whom have received pre-pregnancy care. We also have the additional benefit of having a large cohort of women with normal glucose tolerance for comparison.

Lapolla et al. [18] published a paper in 2007 comparing outcomes with diabetes and the general Italian population. They recorded similar numbers of women receiving pre-pregnancy counseling to our group and similar outcomes for women with diabetes but they compared outcomes with the Italian population in general not a matched control population. Hillman et al. [3] published a paper from Spain comparing outcomes for type 1 and type 2 diabetes. They had similar levels of glycemic control to our groups however they recorded less large for gestational age deliveries, neonatal respiratory distress syndrome and cesarean sections in their type 2 pregnancies compared to the type 1 women. They recorded a higher rate of premature deliveries in both groups than our group. There is no information given about the ethnic origins of the group so this may explain the difference with regards to baby size. There was no information about folic acid use and no control group in this study. Roland et al. [19] found a higher rate of serious adverse outcome in those with type 2 than type 1 diabetes.

There has been one meta-analysis published comparing outcomes for diabetes subtypes [20]. This comprised 33 studies, with varying ethnicities and study numbers. They found overall no difference in outcomes, except a lower rate of cesarean section amongst women with type 2 diabetes. They were unable to find any variables as significant predictors of poor outcome but the author comments that the posthoc analysis had low statistical power given only a small number of the original studies were included.

Since this meta-analysis several recent studies have also compared outcomes between these two groups. Knight et al. [21] in the USA in 2012 and Murphy et al. [22] in the UK 2011 both found results similar to this study, with better outcomes for women with T2DM. However Handisurya and colleagues [23] from Austria in 2011 and Wahabi et al. [24] from Saudi Arabia in 2012 analysed 200 and 112 pregnancies respectively and both reported outcomes that were similar for T1DM and T2DM.

Many studies have shown a high incidence of hypertensive disorders of pregnancy amongst women with both types of diabetes [1,2]. Our study showed that the problem of PET occurs more in those with T1DM compared to controls but in T2DM and matched controls it occurred with similar frequency (8% of cases). This highlights the importance of using a control population as when compared with our general pregnant population PET rates differ between T2DM and controls. Gestational hypertension occurred with similar frequency in both T1 and T2 pregnancies.

Cesarean section deliveries are common in modern obstetric practice. They are not however without complication and surgical complications are more common in women with a higher body mass index [16] and those with diabetes [25]. We saw an overall increase in cesarean section rate, with more emergency cesarean sections completed for those with T1DM and more elective cesarean sections completed in both groups compared to controls. The higher emergency section rate in those with T1DM is likely a reflection of more congenital malformations, greater number of babies >4 kg, and more polyhydramnios and PET. This difference is important for accurately counseling women of their chances of requiring operative delivery.

Heavier birth weights have been shown amongst babies of women with diabetes. Heavier birth weight and macrosomia are associated with short and long term health complications [26,27]; birth trauma, shoulder dystocia, higher rate of cesarean section and in the long term obesity and dysglycemia [28]. The mean birth weights in our groups were similar, but there was a higher prevalence of birth weight greater than 4 kg in babies of mothers with type 1 diabetes compared to matched controls. This did not translate into any significant difference in rates of shoulder dystocia, however numbers were too small to prove a difference in this relatively rare complication.

Birth outcomes were less favorable for babies of women with type 1 diabetes, with a higher stillbirth a non-significantly higher congenital malformation rate [17,29]. This may be as a direct result of a longer duration of diabetes with more micro-vascular disease and less optimal glycemic status at the start of pregnancy with only 41% having an HbA1C <7% in trimester 1. Micro-vascular disease has been demonstrated to compromise placental circulation and to increase risk of stillbirth and preeclampsia [9,13]. These outcomes are essential knowledge for physicians but again are important for accurately counseling women regarding the risks of diabetes on their pregnancy. These outcomes are in spite of almost 40% of women attending pre-pregnancy care. Pre-pregnancy care has been shown to lower morbidity and mortality for pregnancies complicated by diabetes [12].

The link between glycemic control and pregnancy outcome is strong. However what is less strong is the knowledge about what level of glycemic control is necessary to minimize poor outcome and if this level of glycemic control is the same for those with T1DM and T2DM. We saw that the HbA1C values achieved by women with T2DM were lower throughout the whole pregnancy period than it is for those with T1DM. The mean HbA1C values at which poor outcomes occurred were also lower for women with T2DM than they were for women with T1DM. The exact reason for this and whether it could have clinical practice implications is as yet unclear. Our hypothesis is that these are outcomes have multiple contributors beyond glycemic control; for example obesity, gestational weight gain, hypertension, ethnic origin. For those with T1DM the microvascular burden of a longer duration of diabetes likely also has a significant role in these worse outcomes [30]. Although further studies are necessary, it is important too to comment that high insulin doses required for tight diabetes control may result in higher weight gain during pregnancy and this might be adding insult to injury [1,31].

## Conclusions

Overall the known outcomes associated with pregnancies complicated by diabetes were seen in our study in women with both type 1 and type 2 diabetes with a strong relationship to glycemia. However poorer neonatal outcomes occurred more commonly in babies of mothers with type 1 diabetes. Early or pre-pregnancy BMI, gestational weight gain and attendance at pre-pregnancy care have also been shown to be significant contributors to pregnancy outcome and are factors that should be addressed. The higher prevalence of these risk factors in T2DM, especially high BMI, may counteract the positive effect of better glycemic control, resulting in pregnancy outcomes that are not dramatically better than that of T1DM. In our study there was a trend towards less preeclampsia, neonatal hypoglycemia, neonatal unit care and macrosomia in T2DM than in T1DM pregnancies. On the other hand gestational hypertension, elective cesarean section, stillbirth and congenital abnormalities were common problems to both types of diabetes. While the goals of treatment of diabetes during pregnancy are similar for both types, individual factors should be considered when estimating risk of poor outcomes, in order to accurately counsel patients.
